# Predicting pathogenic non-coding SVs disrupting the 3D genome in 1646 whole cancer genomes using multiple instance learning

**DOI:** 10.1038/s41598-021-93917-y

**Published:** 2021-07-13

**Authors:** Marleen M. Nieboer, Luan Nguyen, Jeroen de Ridder

**Affiliations:** 1 Center for Molecular Medicine, University Medical Center Utrecht, 3584 CG Utrecht, The Netherlands; 2 Oncode Institute, Utrecht, The Netherlands

**Keywords:** Computational biology and bioinformatics, Computational models, Data integration, Data processing, Genome informatics, Machine learning

## Abstract

Over the past years, large consortia have been established to fuel the sequencing of whole genomes of many cancer patients. Despite the increased abundance in tools to study the impact of SNVs, non-coding SVs have been largely ignored in these data. Here, we introduce svMIL2, an improved version of our Multiple Instance Learning-based method to study the effect of somatic non-coding SVs disrupting boundaries of TADs and CTCF loops in 1646 cancer genomes. We demonstrate that svMIL2 predicts pathogenic non-coding SVs with an average AUC of 0.86 across 12 cancer types, and identifies non-coding SVs affecting well-known driver genes. The disruption of active (super) enhancers in open chromatin regions appears to be a common mechanism by which non-coding SVs exert their pathogenicity. Finally, our results reveal that the contribution of pathogenic non-coding SVs as opposed to driver SNVs may highly vary between cancers, with notably high numbers of genes being disrupted by pathogenic non-coding SVs in ovarian and pancreatic cancer. Taken together, our machine learning method offers a potent way to prioritize putatively pathogenic non-coding SVs and leverage non-coding SVs to identify driver genes. Moreover, our analysis of 1646 cancer genomes demonstrates the importance of including non-coding SVs in cancer diagnostics.

## Introduction

On average, cancer develops through the accumulation of 4–5 driver mutations^1^. The implications of characterizing these mutations per cancer genome for developing novel anti-cancer therapies are undoubtedly large. Over the recent years, efforts such as the Cancer Gene Census (CGC) have been set up to catalogue all known genes that have been implicated by cancer-driving mutations^2^. Furthermore, a myriad of computational algorithms have been designed to predict the pathogenicity of mutations^3–10^. However, until now the majority of these studies have focused on mutations occurring in the coding part of the genome, while it is becoming increasingly clear that non-coding mutations may also drive cancer initiation and progression^11^.

Elucidating the pathogenic effect of non-coding single-nucleotide variants (SNVs) is under very active study^12–16^, and despite the fact that this is a challenging computational task, prediction results have been gradually improving. Relatively straightforward approaches are based on burden testing^17,18^, wherein elevated mutation densities point to mutations that are under positive selective pressure. However, these statistics-based approaches are not suitable for mutations with low recurrence across cancer patients, which is typically true for non-coding structural variants (SVs), as was recently demonstrated in a Pan-Cancer Analysis of Whole Genomes (PCAWG) study^19^. More recent work therefore focuses on using machine learning to identify patterns in genomic features overlapping and surrounding the SNVs, such as enhancers, histone modifications or transcription factor binding information^12,13^. Despite this progress, almost no methods exist that allow identification of likely pathogenic non-coding SVs. This is counterintuitive, as the impact of somatic SVs (e.g. insertions, deletions, duplications, inversions and translocations) in terms of the number of affected bases far surpasses that of somatic SNVs. For this reason, elucidating the role of non-coding SVs is important for understanding cancer development and may prove to be indispensable for whole genome sequencing (WGS)-based patient reporting.

Although in many cases the exact mechanism through which non-coding SVs cause cancer remains unclear, recent studies have shown that non-coding SVs may exert a pathogenic effect by disrupting the boundaries of Topologically Associated Domains (TADs). TADs are structures in the 3D genome in which DNA interacts more frequently with each other than with DNA outside of the TAD^20^. TADs are separated by boundaries across which interactions are much scarcer. Together, these structures maintain interactions between genes and regulatory elements such as enhancers. TADs are believed to be the result of a process called loop extrusion, in which DNA is pulled through a ring of cohesin until it is blocked by CCCTC-binding factor (CTCF)^21^. This theory is supported by the observation that convergent CTCF motifs were found to be enriched at the boundaries of TADs^22^. Non-coding SVs were found to be capable of causing congenital abnormalities^23–27^ and cancer^28–32^ by disrupting TAD boundaries and thereby enabling novel interactions to form between genes and regulatory elements. However, methods that exploit this principle for somatic SV prioritization or classification have only recently been introduced and remain scarce^33,34^.

While there are sufficient indications that disrupting TAD boundaries can be pathogenic, less is known about the role of disrupting CTCF-mediated chromatin loops that are formed inside of TADs. Previous work suggests that somatic SNVs can affect the binding sites of CTCF and thereby have cancer-driving potential^35^. On the other hand, it was found that not all CTCF loops disrupted by germline non-coding SVs equally contributed to the development of congenital phenotypes^36^. It therefore remains an open question whether somatic non-coding SVs exist that exert a pathogenic effect through CTCF loop disruption, but if they do it may be important to supplement non-coding SV prioritization information with CTCF loop data.

State-of-the art non-coding SNV prioritization algorithms are not straightforwardly applied to SVs. It is, for instance, much more difficult to define a suitable representation of the large number of interactions that may be altered by SVs. Moreover, no ’ground truth’ labels on the pathogenicity of non-coding SVs are available that can be used for training. To this end, we previously proposed a Multiple Instance Learning (MIL)-based approach, called svMIL^34^. A common analogy to explain MIL is the problem of a number of keychains and a door that is opened by one specific key^37^. Without knowing beforehand which key opens the door, the goal is to distinguish the keychains containing at least one key that opens the door (positive keychains or ’bags’) from keychains that do not open the door (negative keychains or bags). As a keychain may contain a variable number of keys (’instances’), representing all keys in a single feature matrix is not trivial. Instead, in MIL, each key is individually described with features such as the length or shape of the key. The challenge for MIL-based classifiers is to separate positive bags (keychains) from negative bags (keychains) within the MIL feature space, which can for example be achieved by mapping the bags to a new feature space in which a regular classifier can be trained^38^.

**Figure 1:**
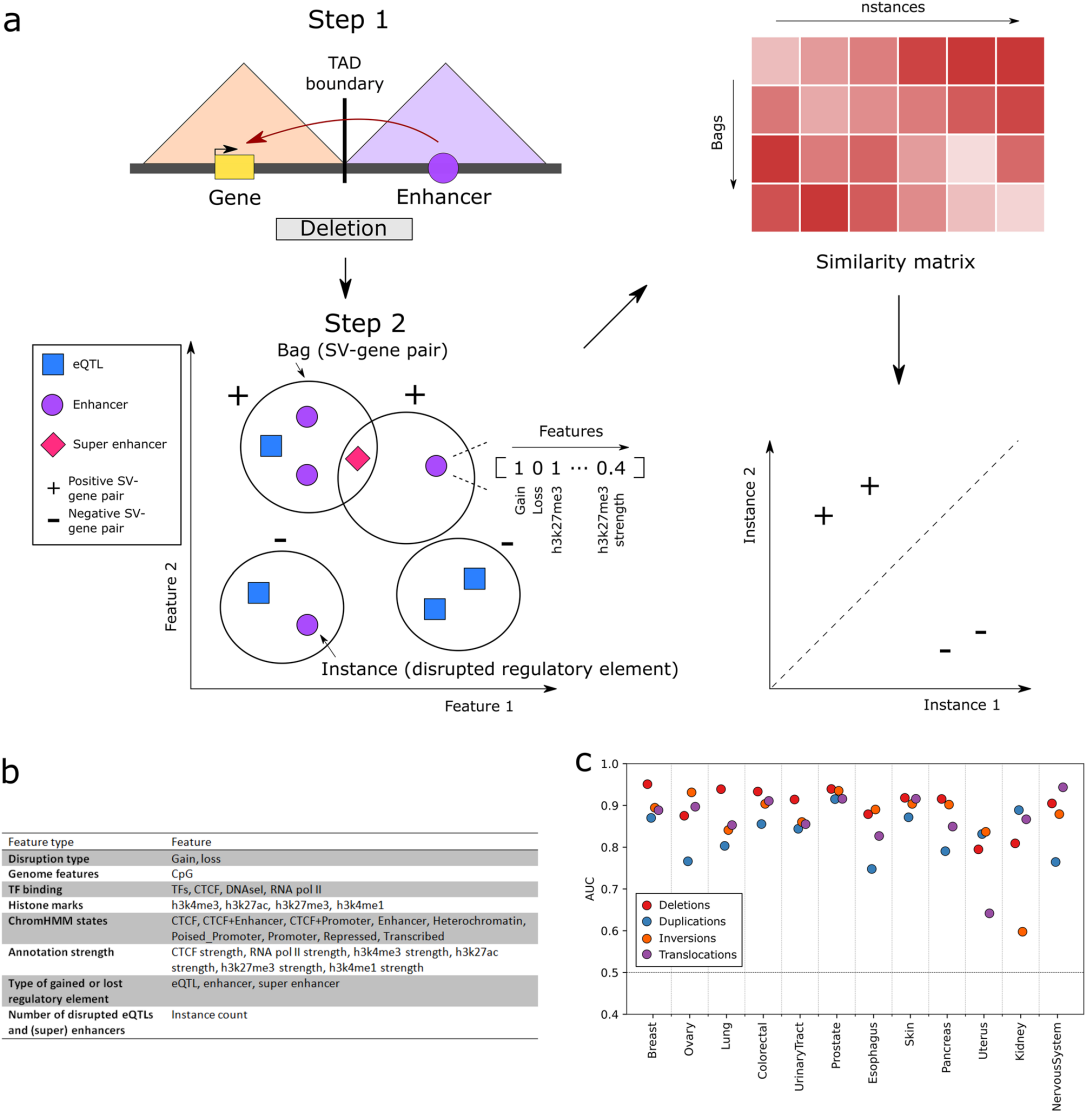
Overview of the svMIL2 method and performance. **(a)** svMIL2 methodology. From disrupted TADs, pairs are identified between SVs and genes disrupted due to gained or lost regulatory elements. These SV-gene pairs are modeled as bags (keychain), in which the regulatory elements (eQTLs, enhancers or super enhancers) that the gene gained or lost due to the SV are instances (keys). Instances are described with features such as histone marks (see panel **b**). A similarity score is constrcuted between bags and instances by computing the absolute distance from the mean instance of each bag to all other instances. The resulting similarity matrix is used as input to a random forest model to classify bags. **(b)** All features used in the svMIL2 model to describe instances, grouped by feature category. **(c)** Performance in AUC of the svMIL2 model on 12 cancer types from the HMF dataset.

In svMIL, we formulated the prediction of pathogenic non-coding SVs as a MIL problem, wherein SV-gene pairs are considered as bags and the regulatory elements as instances (Fig 1a). Labels are obtained by leveraging patient matched gene expression data. Together, this representation enables identification of putatively pathogenic TAD boundary-disrupting non-coding SVs by learning the characteristics of disrupted interactions between genes and regulatory elements. Here, we extend upon this framework and improve the svMIL algorithm, which was originally tested on a maximum of 162 breast cancer patients and 70 ovarian cancer patients, to scale to larger datasets. We additionally use feature selection to improve the AUC by around 0.1 to an average of 0.86 in 313 breast cancer patients. We apply the improved svMIL algorithm, svMIL2, to characterize pathogenic non-coding SVs across 12 cancer types. For this purpose, we leverage a high-quality pan-cancer dataset from the Hartwig Medical Foundation (HMF)^39^, which consists of 1646 uniformly processed high-depth ($>90$x) metastatic tumor samples along with paired transcriptional profiling data. The availability of same-sample whole-genome sequencing (WGS) and RNA-sequencing data across many cancer types has already resulted in a number of novel studies^40–43^, and likewise makes this dataset extremely suitable for this study.

In this work, we show that svMIL2 can confidently predict pathogenic TAD boundary-disrupting non-coding SV candidates across all cancer types, revealing that especially ovarian and pancreatic cancer appear to be more strongly driven by non-coding SVs than other cancers. Furthermore, non-coding SVs frequently disrupt active (super) enhancers in open chromatin regions uniformly across cancer types, which supports our previous findings in breast cancer^34^. Altogether, these findings indicate a common mechanism by which non-coding SVs may cause cancer.

Additionally, we explore the impact of non-coding SVs disrupting intra-TAD CTCF loops rather than TAD boundaries. Although we find that gene expression can be altered through mechanisms similar to TAD boundary disruptions in breast cancer, the frequency at which these events occur is low, confirming previous findings^36^. However, these initial results suggest that investigating the disruption of intra-TAD chromatin loops may be highly relevant in future studies to obtain a complete overview of cancer development and progression.

## Results

### Multiple instance learning effectively predicts pathogenic non-coding SVs

svMIL predicts pathogenic TAD boundary-disrupting non-coding SVs in 2 steps: first predicting candidate pairs of somatic non-coding SVs and disrupted genes, and then applying machine learning to identify the pairs that are pathogenic (Fig 1a, see “Methods” for more details). In step 1, for every SV overlapping a TAD boundary, derivative TADs are constructed in which the disrupted interactions between genes and regulatory elements are modeled (Fig S1). Genes that gain or lose at least 1 regulatory element and the disrupting SV are considered a pair. In step 2, we learn pathogenic SV-gene pairs using a MIL model. Each SV-gene pair is defined as a bag containing the gained or lost eQTLs, enhancers and super enhancers as instances. Every instance is assigned a feature vector (Fig 1b) describing if the instance was gained or lost, which histone marks (h3k4me3, h3k27me3, h3k27ac, h3k4me1), chromatin states (CTCF, CTCF + enhancer, CTCF + promoter, promoter, poised promoter, heterochromatin, repressed, transcribed), transcription factor binding profiles (DNAse I hypersensitivity sites, RNA polymerase II, CTCF, transcription factor binding sites) and CpG islands it overlaps with, the peak intensity (used to indicate strength of the element) of these regulatory elements where available (histone marks, RNA polymerase II), the type of the regulatory element (eQTL, enhancer or super enhancer) and the number of regulatory elements disrupted by the SV (instance count) (see Table S1 for data sources).

To obtain a final classifier, we used the MILES approach with a random forest classifier^38^. In MILES, a feature space is created by computing a bag-to-instance similarity matrix by computing a distance between each bag to all instances, on which a regular classifier can then be trained. Positive bags are expected to have higher similarity to positive instances, but dissimilar to negative instances, resulting in a separation in feature space (Fig 1a). Here, an absolute distance is computed from the mean instance of each bag to all instances.

Bags are labeled positive if the z-score of the expression of the gene in an SV-gene pair to all other patients with no mutation affecting the gene (coding SNV, CNV, SV or non-coding SV) is larger than 1.5 or smaller than −1.5 (i.e. the SV led to altered expression of the paired gene), and negative otherwise.

Model performance is measured using leave-one-patient-out CV, mimicking a scenario in which an unseen patient comes into the clinic. In this CV setting, all SV-gene pairs of one patient are used as testing data, whereas the SV-gene pairs of all other patients are used as training data.

To improve svMIL, we include a rigorous feature selection approach to determine which features optimally benefit the classification result. To this end, we first explored the feature importance in the original model on the breast cancer samples, as this was the cancer type used to infer this model originally. We find that certain features have low variance across instances and do therefore not contribute to classification performance (Fig S2). By removing non-informative features and reducing noise in our instances (see Methods), we further enhance the ability of our previously described svMIL approach to predict pathogenic TAD boundary-disrupting non-coding SVs. Comparing the performance in a leave-one-patient-out CV setting of the original model to the updated model reveals that these improvements yield an increase in AUC of around 0.1 for all SV types except for duplications, which increases by 0.03 (Fig S3). Thus, the methodology of svMIL2 is highly effective at predicting pathogenic non-coding SV-gene pairs.

### svMIL2 can accurately predict driver genes disrupted by non-coding SVs across cancer types

We applied svMIL2 to predict pathogenic non-coding SV-gene pairs in all 12 cancer types from HMF in a leave-one-patient-out CV setting and show that the AUC is consistently high, revealing that our method is also applicable to non-breast cancer data (Fig 1c), even in data with lower sample and SV counts (Fig S4a,b, Table S2). Out of 204 overlapping (100 bp) SVs within different patients, svMIL2 predicts 172 with the same label, showing that our method is robust.

Notably, lower performance is observed for translocations in uterus cancer and for inversions in kidney cancer, which is likely explained by a low sample count and low number of detected pathogenic SVs in these cancers (see Methods and Fig S4c, Table S2). Overall, differences in performance between SV types may be caused by the varying number of SVs of a certain type detected in each cancer.

To maximize the number of correctly identified pathogenic SV-gene pairs, the operating point of each model was individually optimized for the highest recall, requiring a minimum precision of 0.5. In total, 9261 candidate non-coding SV-affected driver genes were identified, ranging between on average 6–35 genes per patient depending on the cancer type (Fig S5a). 346 of the predicted genes are reported in the COSMIC CGC, of which 25 are also annotated to be specific for the respective cancer type (Fig S5b).

11 of the predicted genes have been previously reported as being affected by non-coding SVs, all of which result in significant changes to gene expression compared to non-mutated genes ($\hbox {z} > 1.5$ or $\hbox {z} < -1.5$, see “Methods”). Most notably, we identify a deletion (Fig S6a) and translocation (causing eQTL gains) affecting TP53 in prostate cancer, and an inversion (Fig S6b) and translocation causing ERBB2 to gain eQTLs and a (super) enhancer in ovarian cancer. These genes were reported to be driven by non-coding SVs in these cancer types previously^19^. PTEN (inversion causing gain of an enhancer, super enhancer and eQTL in ovarian cancer), BCL2 (deletion causing gain of an eQTL and enhancer, colorectal cancer), VMP1 (inversion causing gain of an enhancer, super enhancer and eQTL in pancreatic cancer) and LSAMP (translocation causing gain of eQTL in nervous system cancer) were also significant in the same study, albeit in different cancer types.

Other interesting findings include MYB, which is affected by an inversion leading to a (super) enhancer-hijacking event in a colorectal cancer patient, a phenomenon that has previously been observed to occur in ACC as a result of translocations^44^. We also identify a deletion causing GFI1 to gain an eQTL, enhancer and super enhancer in colorectal cancer and an inversion causing a gain of an eQTL in prostate cancer. Enhancer-hijacking was previously demonstated to lead to overexpression of GFI1 in medulloblastoma^45^.

Activation of the proto-oncogene TAL1 was linked to recurrent deletions of a nearby TAD boundary in T-ALL^28^, and we identify potential disruptions of this gene in esophagus cancer (translocation causing gain of eQTL and enhancer) and uterus cancer (translocation causing gain of eQTL). In another study, mutations in the CTCF motif at a TAD boundary nearby NOTCH1 likely resulted in misregulation through novel gene-enhancer interactions^46^. svMIL2 identified an inversion in esophagus cancer causing the gene to gain an eQTL and potentially cause the upregulation of the gene. Finally, recurrently disrupted CTCF sites were observed near FOXC1 in esophagus, gastric and colon adenocarcinomas, and near BCL6 in hepatocellular carcinoma^31^. We identify a deletion causing FOXC1 to gain an eQTL and enhancer in pancreatic cancer, and a duplication resulting in a gain of an eQTL for BCL6 in colorectal cancer.

**Figure 2:**
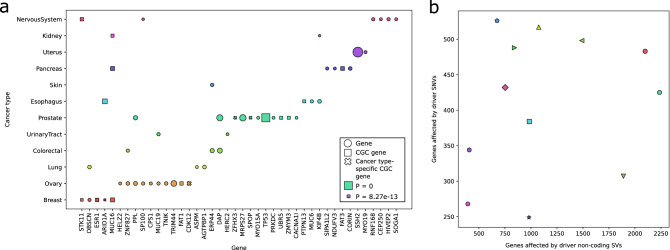
Analysis of predicted pathogenic non-coding SV pairs. **(a)** Genes affected by pathogenic non-coding SVs as identified by svMIL2 with significant driver potential (showing top 50 most significant gene-cancer type pairs). To determine significant driver potential, random gene sets were sampled 10,000 times with the same size as the number of genes with candidate pathogenic non-coding SVs. A t-test was used to compute which of the candidate genes have more driver coding SNVs (snpEff moderate or high impact, filtered for consensus genes driven by SNVs from IntOGen) than expected by random chance. **(b)** Comparison of the number of genes affected by pathogenic non-coding SVs with the number of genes affected by driver SNVs reveals a preference for a different driving mechanism per cancer type.

To validate if these predicted driver genes are significant findings, we determined how frequently they harbor predicted pathogenic SNVs. To this end, we defined the driver potential as the number of driver SNVs affecting the gene across patients within the respective cancer type according to snpEff (moderate or high impact). This list was further filtered for genes driven by SNVs from the IntOGen catalog^47^. Within each cancer type, significance of a gene is assessed by comparing the driver potential to the average driver potential in 10,000 randomly subsampled gene sets of the same size (t-test, Bonferroni corrected). This analysis reveals 112 genes disrupted by non-coding SVs with significant driver potential (Fig 2a, showing the top 50 most significant gene-cancer type combinations. The full list is provided in Table S3). 26 significant genes are also indicated as driver genes by the CGC, of which ESR1, ARID1A, CDK12, ZFHX3 and SPOP are known drivers in breast, ovarian and prostate cancer, respectively. Thus, our model can identify non-coding SVs affecting known driver genes in various cancer types in previously unseen patients.

### The number of pathogenic non-coding SVs varies between cancer types

The highest number of pathogenic non-coding SVs is detected in breast, ovarian and prostate cancer, while only low numbers are identified in uterus and kidney cancer (Fig S4c, Table S2). Although the number of pathogenic non-coding SVs increases with the total number of SVs detected within a cancer type, uterus, nervous system and ovarian cancer have more pathogenic non-coding SVs relative to their total SV count (Fig S4d, Table S2). However, there does not appear to be a clear preference for specific SV types in any cancer type (Fig S7). To determine if certain cancer types may be largely driven by non-coding SVs, we plotted the number of genes affected by at least one predicted pathogenic non-coding SV to the genes with driver SNVs from snpEff and IntOGen as detailed above (Fig 2b). Ovarian and pancreatic cancer stand out as having relatively more pathogenic non-coding SVs than driver SNVs. As tumorigenesis is known to be driven by copy number alterations in these cancer types^48–50^, these findings indicate that many of these events may exert driving effects through disrupting TAD boundaries.

### Pathogenic non-coding SVs disrupt similar regulatory elements across cancer types

**Figure 3:**
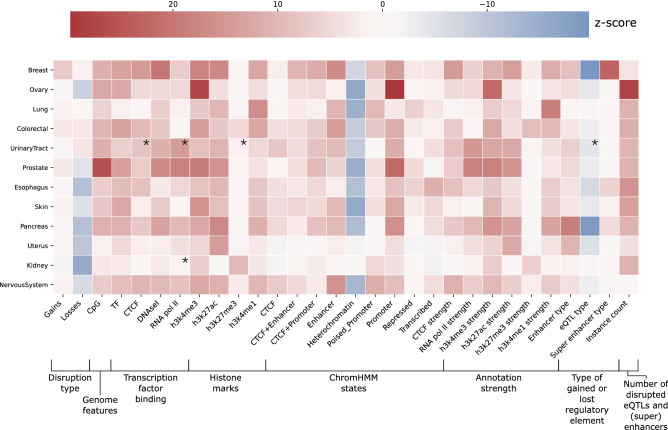
Heatmap showing the instances observed more (red) or less (blue) frequently than expected by random chance in each cancer type. The colors represent the z-score. The asterisks indicate regulatory elements that were missing in a cancer type and for which GM12878 was used as default.

To determine if non-coding SVs exert pathogenicity through similar mechanisms across cancer types, we compared if gained and lost regulatory elements significantly differ between predicted pathogenic SVs and predicted non-pathogenic SVs. For each cancer type, the top 100 instances with highest feature importance were compared to 100 randomly selected instances from predicted non-pathogenic SVs (t-test, Bonferroni correction across all cancer types). Overall, we observe that highly similar regulatory elements are disrupted across cancer types (Fig 3). This is also visible if the affected regulatory elements are split into gains and losses (Fig S8). Interestingly, only breast cancer appears to be driven more by gains than losses of regulatory elements, which is not explained only by a higher number of deletions and duplications (Fig S7) and thus may represent a preferential mechanism to upregulate genes in this cancer type. For kidney and uterus, the overall lower significance is likely explained by a lower number of pathogenic SVs (Fig S4c, Table S2). Across cancer types, we notice a frequent disruption of enhancers and the active enhancer (h3k27ac) mark with high active signal strength (h3k27ac strength). For breast cancer, super enhancers are disrupted. Furthermore, lack of heterochromatin, repressed regions and h3k27me3 (marker of heterochromatin) is frequently observed, while more DNAseI hypersensitivity marks (accessible chromatin) are affected. In conclusion, these patterns indicate that pathogenic non-coding SVs appear to mostly alter active (super) enhancers in open chromatin regions, a mechanism which is recurrently observed across cancer types.

### Tissue-specific regulatory elements are important for classifier performance

**Figure 4:**
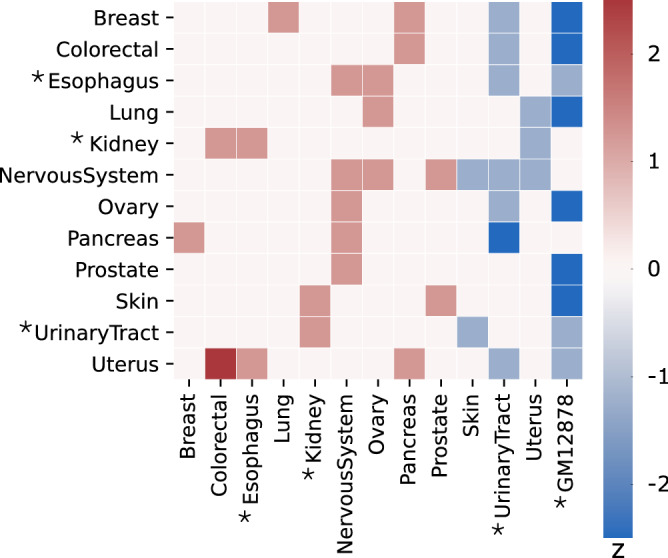
The effect of swapping regulatory data between cancer types on model performance. The z-score is computed by comparing the total AUC difference in a swap across all SV types to the mean of performance differences from the original run to all other swaps, divided by the standard deviation of these differences. Higher z-scores thus mean that the performance is better with data from that tissue type relative to all other tested tissue types in the swap. For example, out of all swaps made, nervous system relatively performs best with data from nervous system, ovary and prostate, while the performance is worst with data from skin, urinary tract and uterus. The asterisks indicate cancer types with some missing data for which GM12878 was used.

As regulatory data may not always be readily available for every tissue, we aimed to assess the impact of selecting less-than-optimal regulatory information on predictive performance. For every cancer type, we ran svMIL2 while swapping all regulatory data with all other cancer types and measured the effect on performance (see Methods). In addition, we compared the performance to a scenario where only data from GM12878 is used, which we use as a default when tissue-specific data is missing. Overall, it appears that the majority of swaps do not significantly alter performance, revealing the overlapping nature of regulatory information between tissue types (Fig 4), which has been noted previously^51^. Using regulatory data from GM12878 and urinary tract are typically poor choices that reduce predictive performance ($\hbox {z} < -1$). As urinary tract misses a lot of tissue-specific data and therefore already uses a lot of data from GM12878 in the original run, this reduction may not be surprising. On the contrary, certain swaps appear to improve performance ($\hbox {z} > 1$). These results may not be unexpected given that our samples consist of metastases, which may no longer necessarily completely represent the tissue of origin. However, as not all samples of our dataset within a cancer type metastasized to the same region, recommending an optimal alternative that will also be suitable for independent data is not trivial. Altogether, these findings are of particular importance for the choice of using GM12878 as a default in case of absent tissue-specific data. While the performance using GM12878 only in place of missing data is reasonable (see Fig 1c, where urinary tract, esophagus and kidney used GM12878 to replace missing data), the possibility of obtaining better AUC with the actual tissue type stresses the importance of generating regulatory datasets for each relevant tissue type.

### Non-coding SVs alter gene expression by disrupting intra-TAD chromatin loops

**Figure 5:**
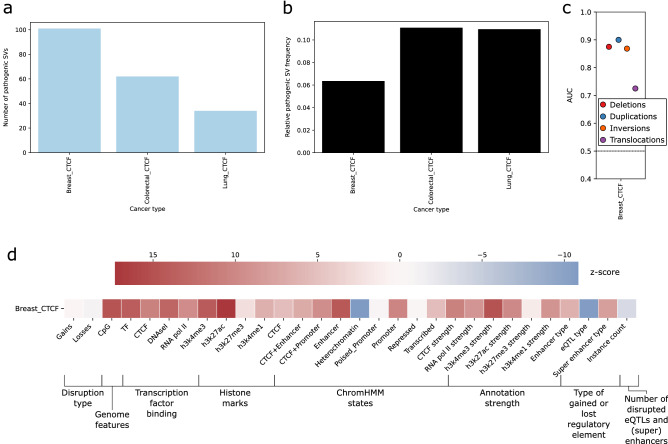
Performance of svMIL2 when intra-chromatin CTCF loops are used in place of TADs. **(a)** Number of pathogenic SV-gene pairs identified to disrupt chromatin loops and **(b)** the percentage of the total SV-gene pairs these comprise. **(c)** AUC of svMIL2 when predicting pathogenic SVs using chromatin loops. **(d)** Regulatory elements more or less frequently disrupted than by random chance for SVs affecting chromatin loops. The colors represent the z-score.

Next, we aimed to determine if SVs disrupting intra-TAD chromatin loops may play a role in cancer. To this end, we ran svMIL2 using chromatin loops predicted by iTAD in place of TAD boundaries. As this software requires cohesin and CTCF peaks as input and these tracks are only available for breast, colorectal and lung cancer, our analysis is limited to those tissue types. By far, most chromatin loops were predicted in breast (breast: 22,113, colorectal: 7522, lung: 9130). In contrast to the TAD-based scenario, the number of SV-gene pairs is far lower (54%, 74% and 83% less in breast, colorectal and lung cancer, respectively), with remarkably fewer pathogenic SV-gene pairs (breast: 101, colorectal: 62, lung: 34) (Fig 5a,b, Fig S4c, Table S2). Taken together, these findings reveal that pathogenic non-coding SVs are less likely to start and end within CTCF loops, but may still alter gene expression.

Due to low counts of candidate SV-gene pairs in colorectal and lung cancer, we could only reliably apply svMIL2 and obtain an AUC in breast cancer, where pathogenic SVs were predicted at high AUC for all SV types (Fig 5c). As the disruption of chromatin loops appears to also frequently result in gains of (super) enhancers in open chromatin regions (Fig 5d, Fig S9), the mechanism by which gene expression is altered is likely similar to that of TAD boundary disruptions.

Out of 94 predicted driver genes affected by SVs through CTCF loop disruption in breast cancer, two are reported as cancer-driving by the CGC. ZNF331 is affected by an inversion, while CHEK2, a well-known germline risk factor for breast cancer^52^, is affected by translocations in four different patients.

In conclusion, we find evidence that non-coding SVs may be capable of altering gene expression in cancer by disrupting intra-TAD chromatin loops, but at a far lower frequency than by the disruption of TAD boundaries, confirming previous findings^36^. However, as our results are limited by the lack of available cohesin measurements across tissues and low sample counts, the importance of intra-TAD loops remains an important topic for future studies.

## Discussion

In this work, we described an improved version of svMIL, svMIL2, to predict pathogenic TAD-boundary and CTCF-loop disrupting non-coding SVs from WGS cancer genomes with paired whole transcriptome sequencing data. We showed that svMIL2 can leverage these data to accurately predict pathogenic non-coding SVs across multiple cancer types. Across all cancer types, putative pathogenic non-coding SVs were predicted to disrupt 9261 genes, 346 of which are known cancer driver genes. Since all validation experiments are carried out through leave-one-patient-out CV, together with identifying non-coding SVs affecting known cancer drivers, these results demonstrate that our method is applicable to identify pathogenic non-coding SVs in a clinical setting where somatic variants of a newly diagnosed patients need to be prioritized. We also observe that the role of pathogenic non-coding SVs, as opposed to driver SNVs, varies between cancers. Despite these differences, non-coding SVs appear to similarly frequently disrupt active (super) enhancers in open chromatin regions in the majority of cancer types, pointing to common mechanisms by which TAD disruptions may be pathogenic. Taken together, these findings indicate that non-coding SVs play an important role in cancer and should be considered in WGS-based cancer diagnostics.

As opposed to the clear impact of disrupting TAD boundaries on the development of cancer, the effects of disrupting intra-TAD chromatin loops are not yet well understood. Using svMIL2, we were able to identify pathogenic non-coding SVs that alter expression of known cancer genes by disrupting CTCF loops in breast cancer. However, the number of candidate pathogenic SV-gene pairs resulting from CTCF loop disruptions is up to tenfold lower than when only TADs are investigated. Therefore, SVs disrupting intra-TAD chromatin loops rather than TAD boundaries may seemingly be less pathogenic, which corresponds with previous experiments performed with germline SVs^36^. However, as we were only able to obtain cohesin and CTCF peak data for breast, colorectal and lung cancer, the actual relevance of chromatin loops may be underreported in this study. Nevertheless, these initial findings point to a potential involvement of disrupting CTCF loops in the development of cancer, and may be a highly interesting avenue for future studies.

While the majority of regulatory information is available in respective tissue types, we found that selecting the most suitable alternative for cases with missing data remains a difficult problem that potentially strongly affects classifier performance. As our dataset is comprised of metastatic cancer data, the reference tissue type may sometimes no longer be well-represented in the cancer at time of sampling, and thus selecting an optimal alternative tissue is not trivial. However, answering these questions will only really become possible once the missing regulatory data have been acquired in the respective tissues. Therefore, our results underscore the importance of completing the catalogue of celltype-specific regulatory information. Such data may also help create a better understanding of the role of SVs in the mitochondrial DNA (mtDNA). Common deletions have been identified in the mtDNA of especially gastric cancers^53^, but the effect of such SVs on regulatory information is difficult to assess as mtDNA is often missing from regulatory datasets. While large-scale efforts to collect these data such as the ENCODE project^54^ are still ongoing, other promising alternatives to acquire these data apply imputation from other cell types, which is performed by methods such as Avocado^51^, ChromImpute^55^ and PREDICTD^56^. However, as imputation with these methods is not yet possible for regulatory data in all tissue types, further research in this field is required.

Furthermore, our method could further benefit from improved SV calls. While our current dataset captures many SVs in the genome, adopting long-read sequencing techniques could improve detection of additional SVs in repetitive regions^57^ and clear up potentially noisy calls. SVs obtained from longer reads can improve the training labels used in svMIL2, as expression of certain genes may be altered due to non-coding SVs but currently remain undetected due to missing calls. Label quality would also benefit from additional patient-matched datasets such as methylation data, which could be used to exclude genes that are deregulated due to methylation rather than non-coding SVs. However, such data is currently too costly to routinely generate for each patient. Similar labeling problems occur when genes are affected by variants of unknown significance or upstream pathway effects, which are difficult to account for. While methods such as DriverNet^58^ or DawnRank^59^ have been shown to improve driver prediction by integrating gene networks with SNV and CNV data, non-coding SVs have not yet been included in these studies. However, as the number of recurrent driver non-coding SVs is smaller than for SNVs or CNVs, as was shown previously^19,34^, the statistical validation applied will need to properly deal with the imbalance in contribution to the driver phenotype between the mutation types.

Although we demonstated that MIL is a suitable approach to identify pathogenic non-coding SVs and previously showed the benefits of using MIL compared to a non-MIL random forest^34^, alternative machine learning approaches may assist in learning about pathogenic non-coding SVs from a different perspective. For example, deep learning-based methods such as DeepSEA^12^ and ExPecto^13^ were recently used to prioritize non-coding SNVs by learning genomic features, such as chromatin states, of the region around the mutation. Such an approach could similarly be used to learn the characteristics of SV breakpoints, or disrupted TAD boundaries. These annotations on a smaller scale could teach us more about the local environment disrupted by non-coding SVs in detail, which is not straightforward with svMIL2.

WGS is rapidly becoming part of the routine diagnostic process of cancer centers. However, since the driving potential of non-coding SVs remains elusive, the vast majority of these costly WGS data remain underutilized. Our proposed svMIL2 model can accurately predict pathogenic non-coding SVs among the typically vast numbers of somatic SVs present in cancer genomes by learning from a combination of WGS, gene expression, TAD boundary and intra-TAD chromatin loop information. As more and more WGS datasets and epigenomics tracks will become available, it can be expected that these predictions will further improve. This will further enable the inclusion of non-coding SVs in WGS-based cancer diagnostic reporting.

## Methods

### Data

Pre-called whole-genome SV, CNV and SNV data and RNA-seq counts were obtained for 1944 cancer patients from the HMF, representing 29 cancer types in total. All variants were called using the HMF pipeline (https://github.com/hartwigmedical/pipeline), as detailed previously^60^. The RNA-seq data was processed using Isofox (https://github.com/hartwigmedical/hmftools/tree/master/isofox). The raw expression read counts were normalized across all patients using the Trimmed Mean of M-values (TMM) method. Cancer types with fewer than 20 samples or with uncertain or varying tissue origin were omitted from analysis, leaving 12 cancer types in total across 1646 patients (breast: 313, ovary: 62, lung: 125, colon/rectum: 393, urinary tract: 118, prostate: 199, esophagus: 53, skin: 216, pancreas: 66, uterus: 26, kidney: 38, nervous system: 37).

For all data collection, hg19 was used as the reference genome. We downloaded CpG islands (across all cell types) from the UCSC genome annotation database. Transcription factors (across all cell types) were collected from the ORegAnno database^61^. ChromHMM states (HMEC) were obtained from Taberlay et al.^62^.

The following regulatory elements were downloaded for the tissue types closest matching the cancer type. A detailed overview of all regulatory data sources can be found in Table S1. eQTLs were downloaded from GTEx v7 (v8 for kidney, converted to hg19 using the UCSC liftover tool)^63^. Enhancers were obtained from JEME^64^. Super enhancers were collected from dbSUPER^65^ and SEdb^66^ (kidney, brain and prostate). TADs were downloaded from the 3D genome browser^67^, using the UCSC liftover tool to convert from GRCh38 to hg19 for colorectal and ovary. CTCF, DNAse I, h3k4me3, h3k27me3, h3k27ac, h3k4me1 and RNA pol II peaks were downloaded from ENCODE^54^.

For each cancer type, regulatory data was selected for the closest matching tissue of origin. GM12878 was selected where tissue-specific regulatory data was missing, as this data type is available for all regulatory data and thus represents a typical baseline. The impact of selecting less-than-optimal tissue types is further explored in the Results and the procedure is detailed below.

### svMIL2 model

svMIL2 follows two steps to identify pathogenic non-coding SVs: identifying genes putatively disrupted by TAD boundary-disrupting non-coding SVs, and using MIL to learn which of these SVs are pathogenic. For full details, please refer to the original svMIL publication^34^.

In step 1, all genes are identified that are putatively affected by non-coding SVs disrupting boundaries of TADs (Fig S1). Only SVs that start and end within TADs are included, requiring at least 1 basepair overlap with the TAD. For each SV type, we determine which regulatory elements (eQTLs, enhancers and super enhancers) are disrupted by the SV. eQTLs have been previously shown to overlap with enhancers that regulate known cancer genes^68^, and are therefore included to account for possibly undiscovered enhancers.

For deletions, all genes in the TAD on one side of the deletion will gain the regulatory elements on the other side of the deletion. Regulatory elements and genes that are overlapped by the deletion itself are not counted as these are not TAD-disrupting events.

For duplications, new TADs are created between the overlapped TAD boundary and the position where this overlapped boundary is re-inserted into the genome. Within this new TAD, genes overlapped by the duplication on one side of the TAD boundary will gain regulatory elements overlapped by the duplication on the other side of the TAD boundary. As no clear consensus exists about how many basepairs of a regulatory element need to be affected to disrupt its function, we require a minimum overlap of 1 basepair.

For inversions, genes lose regulatory elements that are inverted out of the TAD, and gain regulatory elements that are inverted into the TAD. Genes inside the inversion will gain regulatory elements of the TAD that these are inverted in to, and lose regulatory elements that were in the TAD it was inverted out of.

For translocations, we construct a derivative TAD based on the SV orientation in which the new positions of genes and regulatory elements are modeled. Genes gain and lose regulatory elements based on if these are introduced into or removed from the new TAD, respectively.

From these TAD disruptions, a list of SV-gene pairs is constructed containing the regulatory elements that the gene gained or lost as a result of the SV. All genes overlapped (1 basepair) by any coding mutation (SVs, SNVs or CNVs) are excluded to ensure that any effect on the gene is explained only by the non-coding SV. An exception is made for non-coding duplications and inversions, which may overlap the affected gene itself.

In step 2, a MIL model is trained to learn which gains and losses of regulatory elements are characteristic of pathogenic non-coding SVs. Every SV-gene pair is considered a bag, with the disrupted regulatory elements (eQTLs, enhancers and super enhancers) as instances. Each instance is described with a single feature vector. The first two features are binary, indicating if the regulatory element was gained or lost. The next set of features contain either a 0 or 1 depending on if the regulatory element overlaps (minimum 1 bp) with any of the following annotations (Fig 1b): histone marks (h3k4me3, h3k27me3, h3k27ac, h3k4me1), chromHMM states (CTCF, CTCF+enhancer, CTCF+promoter, enhancer, promoter, poised promoter, heterochromatin, repressed, transcribed), transcription factor binding profiles (DNAseI hypersensitivity, RNA polymerase II, CTCF, transcription factor binding sites) and CpG islands. The third set of features uses the peak intensity of these annotations where available to indicate their strength (histone marks, RNA polymerase II, CTCF). Finally, binary features were used to indicate the type of the regulatory element (eQTL, enhancer, super enhancer) and the number of regulatory elements disrupted by this SV in total (instance count). All features were normalized between 0 and 1.

To label the bags (SV-gene pairs) as pathogenic or non-pathogenic, a z-score was computed from the gene expression to all patients without a disruption to the gene (e.g. coding SV, SNV, CNV or non-coding SV). Bags with $\hbox {z} > 1.5$ or $\hbox {z} < -1.5$ were labeled positive, and negative otherwise, which was determined to be the optimal threshold in the previous version of svMIL^34^. Negative bags were randomly subsampled to the number of positive bags to obtain class balance.

A final classifier was obtained by applying the MILES approach^38^. In MILES, a standard feature space is constructed by computing a similarity matrix between the bags and instances. Here, we computed the absolute distance from the mean instance of the bags to all instances. In this space, a random forest was trained to obtain a final classifier. A model was constructed for each SV type separately. All performances were measured using a leave-one-patient-out CV, which models a scenario in which an unseen patient would come into the clinic.

### Using svMIL2

svMIL2 takes VCF files containing SVs per patient as input and generates SV-gene pairs based on TAD boundary disruption as detailed above. SV-gene pairs overlapped by coding SNVs, CNVs or SVs are filtered out. SNV files should be provided as VCF files per patient. For CNVs, a tab-delimited file is expected per patient containing the genes and their copy numbers. SV-gene pairs of which the gene has a copy number below 1.7 or above 2.3 are omitted from further analysis. Bags (SV-gene pairs) are labeled for MIL using normalized expression data as described above. To prioritize pathogenic SV-gene pairs, users can either run the MIL in a leave-one-patient-out CV setting, or train the model on one dataset and apply to another. A ranking can be obtained through the classifier probabilities assigned to each bag. A step-by-step tutorial for using svMIL2 is available on GitHub (see Data availability).

### Feature selection to improve model performance

To improve the predictive performance of svMIL, we aimed to improve the quality of features through feature selection. Feature importance was assessed by computing the variance of a feature across all instances of the breast cancer samples (Fig S2). Certain features that were present in the original model (Hi-C, h3k9me3, h3k36me3, chromHMM repeat regions and enhancer, h3k9me3 and h3k36me3 strength) contained low variance (log(variance) $< -10$) and therefore did not contribute to the distinction between positive and negative instances, and were thus omitted.

### Improving method accuracy by increasing the number of high-quality instances

To increase the number of informative instances in the model, the eQTL p-value stringency threshold was increased from 5e−8 to 0.05. To account for the resulting increased computational load, all eQTLs, histone marks, and transcription factor (TF) binding sites were binned using a 1 kb sliding window.

To account for increased memory consumption resulting from a larger number of SV-gene pairs, bags of each SV type were randomly subsampled if their count exceeded 700, which did not significantly reduce performance on the breast cancer samples for all SV types but inversions, for which the AUC is lowered slightly (Fig S10).

### Swapping regulatory elements between cancer types

The effect on performance when swapping regulatory data between cancer types was measured by computing the absolute difference in AUC between the original run and the swapped run, summed across the models for each SV type. A z-score was computed by comparing this summed difference to the mean and standard deviation of the summed differences of all swaps made for that cancer type. Thus, a higher z-score indicates a better performance with that tisue type relative to all other tested tissue types in the swap. For visualization purposes, z-scores were quantized to indicate non-significant effect $(-1< \hbox {z} < 1)$, significant effect ($-2< \hbox {z} < -1$ and $1< \hbox {z} < 2$), and highly significant effect ($\hbox {z} < -2$ and $\hbox {z} > 2$).

### Running svMIL2 with CTCF loops instead of TAD boundaries

Intra-TAD chromatin loops were predicted using iTAD^69^. Due to the limited availability of cohesin peak data, predictions were limited to tissues for which both cohesin and CTCF peaks were available (breast, colorectal, lung). For cohesin, RAD21 TF ChIP-seq peaks were downloaded for MCF-7 (breast), HCT-116 (colorectal) and A549 (lung). For CTCF, the files listed in Table S1 were used. To predict pathogenic SV-gene pairs, svMIL2 was run using the predicted intra-TAD chromatin loops in place of TAD boundaries.

## Supplementary Information

Supplementary Figures.

Supplementary Table S1.

Supplementary Table S2.

Supplementary Table S3.

## Data Availability

All (processed) WGS and RNA-sequencing data were provided by the Hartwig Medical Foundation under data request DR-104. This publication and the underlying study have been made possible partly on the basis of the data that Hartwig Medical Foundation and the Center of Personalised Cancer Treatment (CPCT) have made available to the study. All code and processed feature data is publicly available at https://github.com/UMCUGenetics/svMIL/. On GitHub a manual can be found reproducing all paper figures and running svMIL2 on a different dataset.

## References

[1] Campbell (2020). Pan-cancer analysis of whole genomes. Nature.

[2] Sondka Z (2018). The COSMIC cancer gene census: Describing genetic dysfunction across all human cancers. Nat. Rev. Cancer.

[3] Cingolani P (2012). A program for annotating and predicting the effects of single nucleotide polymorphisms. SnpEff. Fly.

[4] Sim N-L (2012). SIFT web server: Predicting effects of amino acid substitutions on proteins. Nucleic Acids Res..

[5] Adzhubei IA (2010). A method and server for predicting damaging missense mutations. Nat. Methods.

[6] Rogers MF (2018). FATHMM-XF: Accurate prediction of pathogenic point mutations via extended features. Bioinformatics.

[7] Rentzsch P, Witten D, Cooper GM, Shendure J, Kircher M (2019). CADD: Predicting the deleteriousness of variants throughout the human genome. Nucleic Acids Res..

[8] Flygare S (2018). The VAAST variant prioritizer (VVP): Ultrafast, easy to use whole genome variant prioritization tool. BMC Bioinform..

[9] Ganel, L., Abel, H. J. & Hall, I. M. SVScore: An impact prediction tool for structural variation. *Bioinformatics* btw789. 10.1093/bioinformatics/btw789 (2016). 10.1093/bioinformatics/btw789 PMC5408916 28031184

[10] Dahary D (2019). Genome analysis and knowledge-driven variant interpretation with TGex. BMC Med. Genomics.

[11] Khurana E (2016). Role of non-coding sequence variants in cancer. Nat. Rev. Genet..

[12] Zhou J, Troyanskaya OG (2015). Predicting effects of noncoding variants with deep learning-based sequence model. Nat. Methods.

[13] Zhou J (2018). Deep learning sequence-based ab initio prediction of variant effects on expression and disease risk. Nat. Genet..

[14] Umer HM (2016). A significant regulatory mutation burden at a high-affinity position of the CTCF motif in gastrointestinal cancers. Hum. Mutat..

[15] Mularoni L, Sabarinathan R, Deu-Pons J, Gonzalez-Perez A, López-Bigas N (2016). OncodriveFML: A general framework to identify coding and non-coding regions with cancer driver mutations. Genome Biol..

[16] Hornshøj H (2018). Pan-cancer screen for mutations in non-coding elements with conservation and cancer specificity reveals correlations with expression and survival. npj Genom. Med..

[17] Dees ND (2012). MuSiC: Identifying mutational significance in cancer genomes. Genome Res..

[18] Tamborero D, Gonzalez-Perez A, Lopez-Bigas N (2013). OncodriveCLUST: Exploiting the positional clustering of somatic mutations to identify cancer genes. Bioinformatics.

[19] Rheinbay E (2020). Analyses of non-coding somatic drivers in 2,658 cancer whole genomes. Nature.

[20] Dixon JR (2012). Topological domains in mammalian genomes identified by analysis of chromatin interactions. Nature.

[21] Fudenberg G (2016). Formation of chromosomal domains by loop extrusion. Cell Rep..

[22] Sanborn AL (2015). Chromatin extrusion explains key features of loop and domain formation in wild-type and engineered genomes. Proc. Natl. Acad. Sci..

[23] Giorgio E (2015). A large genomic deletion leads to enhancer adoption by the lamin B1 gene: A second path to autosomal dominant adult-onset demyelinating leukodystrophy (ADLD). Hum. Mol. Genet..

[24] Redin C (2017). The genomic landscape of balanced cytogenetic abnormalities associated with human congenital anomalies. Nat. Genet..

[25] Franke M (2016). Formation of new chromatin domains determines pathogenicity of genomic duplications. Nature.

[26] Lupiáñez DG (2015). Disruptions of topological chromatin domains cause pathogenic rewiring of gene-enhancer interactions. Cell.

[27] Zhang X (2018). Local and global chromatin interactions are altered by large genomic deletions associated with human brain development. Nat. Commun..

[28] Hnisz D (2016). Activation of proto-oncogenes by disruption of chromosome neighborhoods. Science.

[29] Weischenfeldt J (2017). Pan-cancer analysis of somatic copy-number alterations implicates IRS4 and IGF2 in enhancer hijacking. Nat. Genet..

[30] Valton A-L, Dekker J (2016). TAD disruption as oncogenic driver. Curr. Opin. Genet. Dev..

[31] Akdemir KC (2020). Disruption of chromatin folding domains by somatic genomic rearrangements in human cancer. Nat. Genet..

[32] Dixon JR (2018). Integrative detection and analysis of structural variation in cancer genomes. Nat. Genet..

[33] Huynh L, Hormozdiari F (2019). TAD fusion score: Discovery and ranking the contribution of deletions to genome structure. Genome Biol..

[34] Nieboer MM, de Ridder J (2020). svMIL: Predicting the pathogenic effect of TAD boundary-disrupting somatic structural variants through multiple instance learning. Bioinformatics.

[35] Liu EM (2019). Identification of cancer drivers at CTCF insulators in 1,962 whole genomes. Cell Syst..

[36] Despang A (2019). Functional dissection of the Sox9-Kcnj2 locus identifies nonessential and instructive roles of TAD architecture. Nat. Genet..

[37] Dietterich (1997). Solving the multiple instance problem with axis-parallel rectangles. Artif. Intell..

[38] Chen, Y., Bi, J., & Wang, J. MILES: Multiple-instance learning via embedded instance selection. *IEEE Trans. Pattern Anal. Mach. Intell.* **28**, 1931–1947. 10.1109/TPAMI.2006.248 (2006). 10.1109/TPAMI.2006.248 17108368

[39] Priestley P (2019). Pan-cancer whole-genome analyses of metastatic solid tumours. Nature.

[40] Angus L (2019). The genomic landscape of metastatic breast cancer highlights changes in mutation and signature frequencies. Nat. Genet..

[41] Degasperi A (2020). A practical framework and online tool for mutational signature analyses show intertissue variation and driver dependencies. Nat. Cancer.

[42] Christensen S (2019). 5-Fluorouracil treatment induces characteristic T$$>$$G mutations in human cancer. Nat. Commun..

[43] Samsom, K. G. *et al.* Driver mutations occur frequently in metastases of well-differentiated small intestine neuroendocrine tumours. *Histopathology* his.14252. 10.1111/his.14252 (2020). 10.1111/his.14252 32931025

[44] Drier Y (2016). An oncogenic MYB feedback loop drives alternate cell fates in adenoid cystic carcinoma. Nat. Genet..

[45] Northcott PA (2014). Enhancer hijacking activates GFI1 family oncogenes in medulloblastoma. Nature.

[46] Ji X (2016). 3D chromosome regulatory landscape of human pluripotent cells. Cell Stem Cell.

[47] Martínez-Jiménez F (2020). A compendium of mutational cancer driver genes. Nat. Rev. Cancer.

[48] Bhattacharya A (2020). Transcriptional effects of copy number alterations in a large set of human cancers. Nat. Commun..

[49] McGrail DJ (2018). Multi-omics analysis reveals neoantigen-independent immune cell infiltration in copy-number driven cancers. Nat. Commun..

[50] Lu S, Ahmed T, Du P, Wang Y (2017). Genomic variations in pancreatic cancer and potential opportunities for development of new approaches for diagnosis and treatment. Int. J. Mol. Sci..

[51] Schreiber J, Durham T, Bilmes J, Noble WS (2020). Avocado: A multi-scale deep tensor factorization method learns a latent representation of the human epigenome. Genome Biol..

[52] Apostolou P, Papasotiriou I (2017). Current perspectives on CHEK2 mutations in breast cancer. Breast Cancer Targets Ther..

[53] Ye F, Samuels DC, Clark T, Guo Y (2014). High-throughput sequencing in mitochondrial DNA research. Mitochondrion.

[54] Dunham (2012). An integrated encyclopedia of DNA elements in the human genome. Nature.

[55] Ernst J, Kellis M (2015). Large-scale imputation of epigenomic datasets for systematic annotation of diverse human tissues. Nat. Biotechnol..

[56] Durham TJ, Libbrecht MW, Howbert JJ, Bilmes J, Noble WS (2018). PREDICTD PaRallel epigenomics data imputation with cloud-based tensor decomposition. Nat. Commun..

[57] Treangen TJ, Salzberg SL (2012). Repetitive DNA and next-generation sequencing: Computational challenges and solutions. Nat. Rev. Genet..

[58] Bashashati A (2012). DriverNet: Uncovering the impact of somatic driver mutations on transcriptional networks in cancer. Genome Biol..

[59] Hou JP, Ma J (2014). DawnRank: Discovering personalized driver genes in cancer. Genome Med..

[60] Nguyen, L., Martens, W. M. J., Van Hoeck, A. & Cuppen, E. Pan-cancer landscape of homologous recombination deficiency. *Nat. Commun.* **11**, 5584. 10.1038/s41467-020-19406-4 (2020). 10.1038/s41467-020-19406-4 PMC7643118 33149131

[61] Lesurf R (2016). ORegAnno 3.0: A community-driven resource for curated regulatory annotation. Nucleic Acids Res..

[62] Taberlay PC, Statham AL, Kelly TK, Clark SJ, Jones PA (2014). Reconfiguration of nucleosome-depleted regions at distal regulatory elements accompanies DNA methylation of enhancers and insulators in cancer. Genome Res..

[63] Aguet (2017). Genetic effects on gene expression across human tissues. Nature.

[64] Cao Q (2017). Reconstruction of enhancer-target networks in 935 samples of human primary cells, tissues and cell lines. Nat. Genet..

[65] Khan A, Zhang X (2016). dbSUPER: A database of super-enhancers in mouse and human genome. Nucleic Acids Res..

[66] Jiang Y (2019). SEdb: A comprehensive human super-enhancer database. Nucleic Acids Res..

[67] Wang, Y. *et al.* The 3D genome browser: A web-based browser for visualizing 3D genome organization and long-range chromatin interactions. *bioRxiv* (2017). 10.1186/s13059-018-1519-9 PMC6172833 30286773

[68] Chen H (2018). A pan-cancer analysis of enhancer expression in nearly 9000 patient samples. Cell.

[69] Matthews, B. J. & Waxman, D. J. Computational prediction of CTCF/cohesin-based intra-TAD loops that insulate chromatin contacts and gene expression in mouse liver. *eLife* **7**. 10.7554/eLife.34077 (2018). 10.7554/eLife.34077 PMC5986275 29757144

