# Supplementary material for: Predicting pathogenic non-coding SVs disrupting the 3D genome in 1646 whole cancer genomes using multiple instance learning
Source: Sci Rep. 2021 Jul 13;11:14411. doi: 10.1038/s41598-021-93917-y (PMC8277903; doi:10.1038/s41598-021-93917-y)
Supplement: Supplementary file 1 — Supplementary Figures. [file 41598_2021_93917_MOESM1_ESM.pdf]

Predicting pathogenic non-coding SVs disrupting the 3D genome in 1646 whole cancer genomes using Multiple Instance Learning

Supplementary Data

Marleen M. Nieboer[1,2], Luan Nguyen[1,2], and Jeroen de Ridder[1,2]

[1]Center for Molecular Medicine, University Medical Center Utrecht, Utrecht, 3584 CG, The Netherlands
[2]Oncode Institute, Utrecht, The Netherlands

1 Supplementary figures

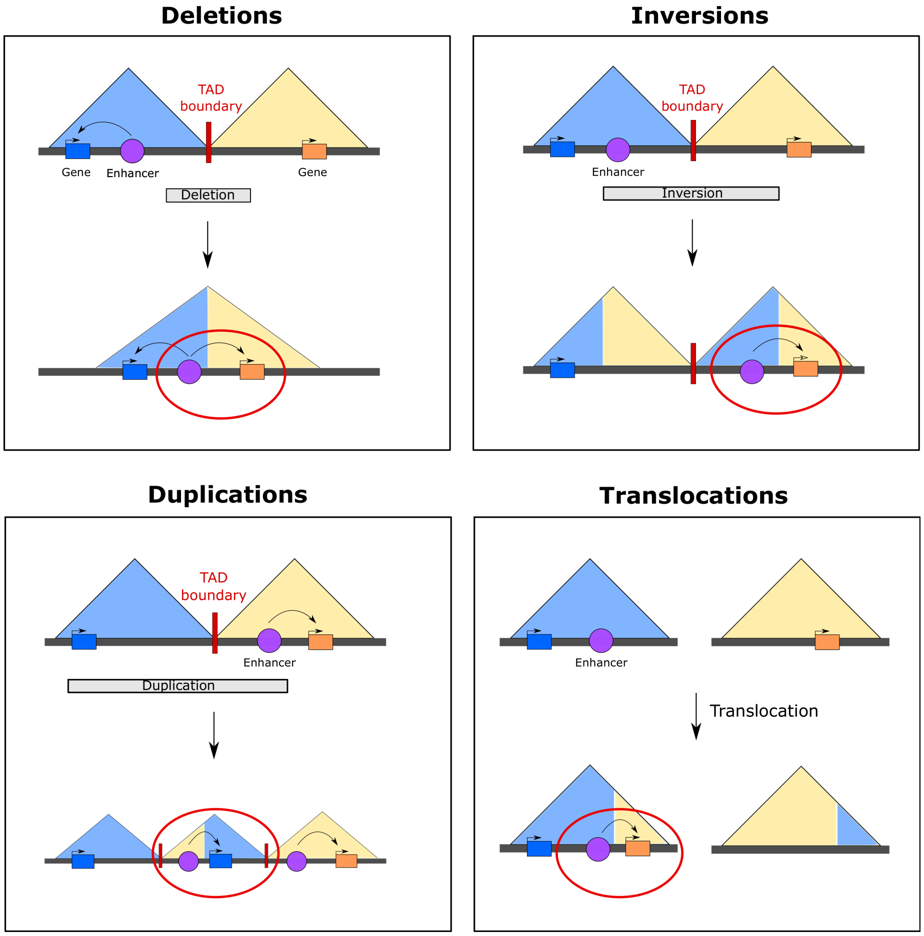

Fig. S1: **Schematic illustration of disruptions of TAD boundaries by non-coding SVs are modeled in svMIL2.** In each example, a gain of interaction with an enhancer is shown. For inversions, the gene in the left TAD also loses potential interactions with the enhancer.

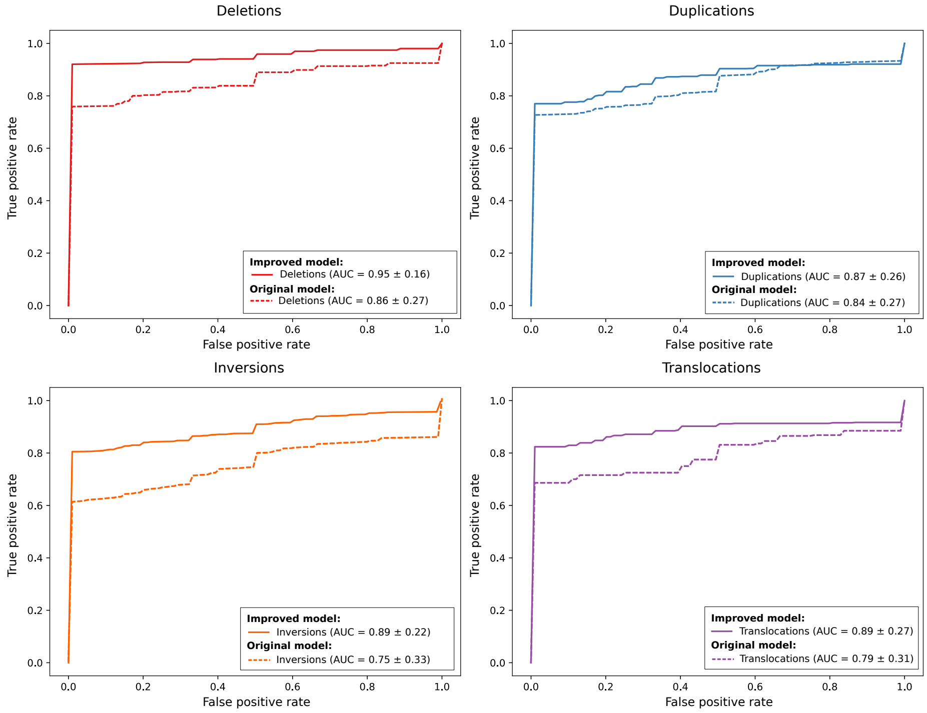

Fig. S2: **Performance ROC curves of svMIL2 compared to the original svMIL on all breast cancer samples in a leave-one-patient-out CV setting per SV type.**

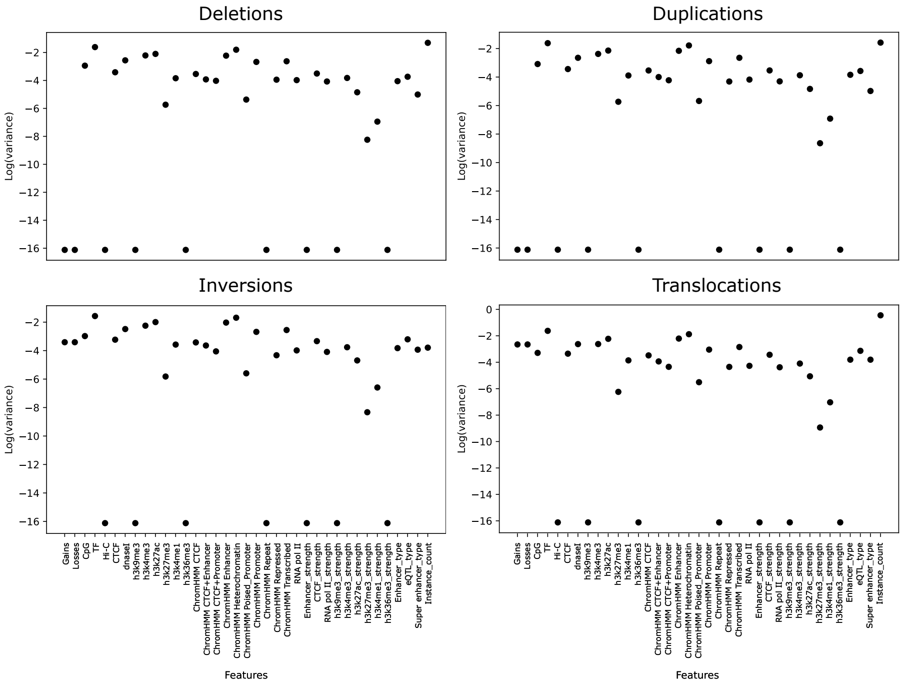

Fig. S3: **Log of the variance across all instances. A margin of 0.00001 was added to variances of 0 to compute the log. Features with variances lower than -10 for all SV types were removed from the model.**

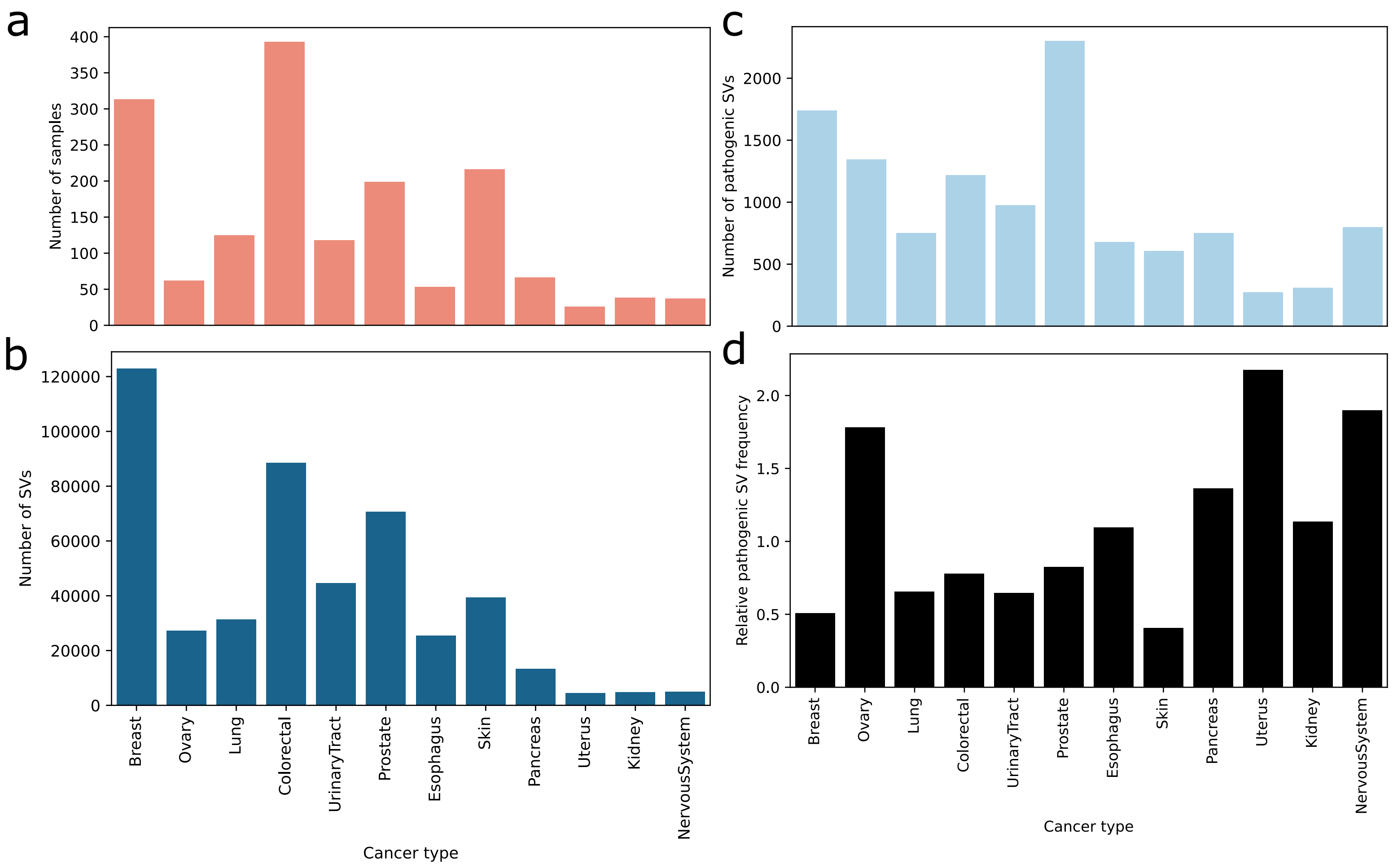

Fig. S4: **Number of (a) samples, (b) SVs and (c) predicted pathogenic SVs in each cancer type. (d) Percentage of predicted pathogenic SVs compared to the total number of SVs across all samples in a cancer type. The numbers in this figure are also provided in Table S2.**

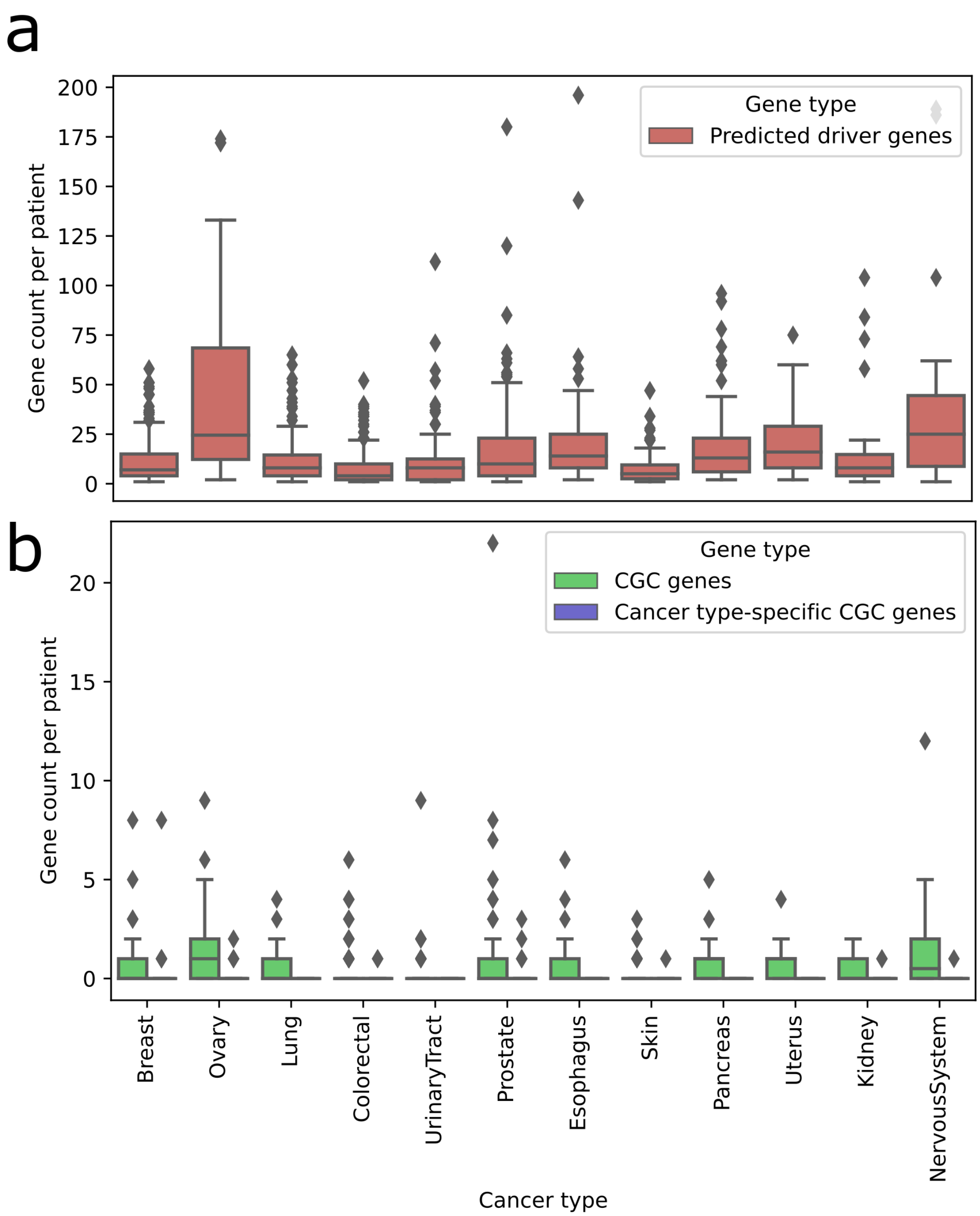

Fig. S5: **Distribution of the number of predicted (a) driver genes and (b) (cancer type-specific) CGC genes disrupted by non-coding SVs across patients.**

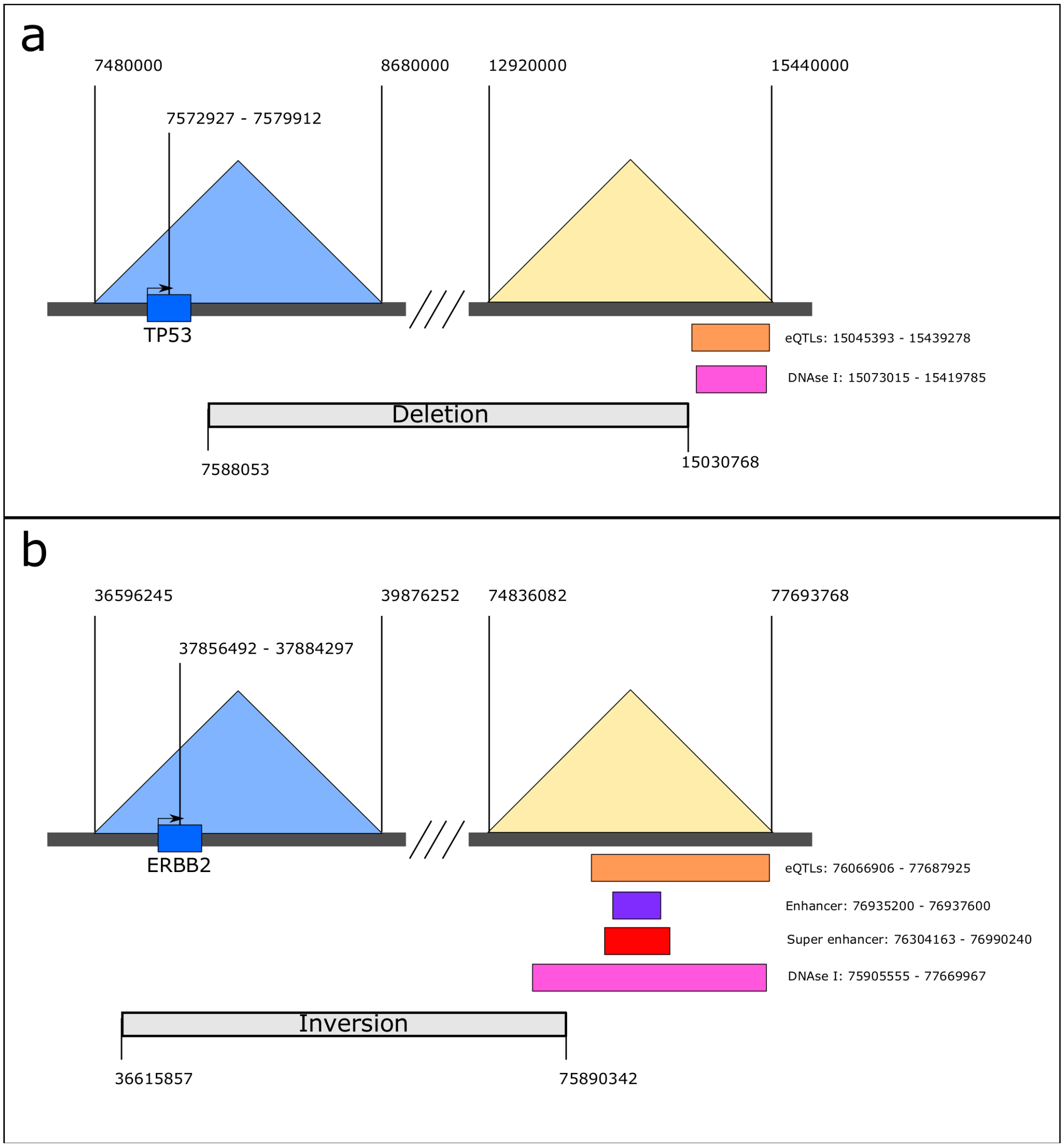

Fig. S6: **Schematic illustration of non-coding SVs exerting possible pathogenic effects on (a) TP53 in a prostate cancer patient and (b) ERBB2 in an ovarian cancer patient.** For ERBB2, the inversion brings the gene into a new TAD where potential new interactions can be formed with a cluster of eQTLs, an enhancer and a super enhancer that are located in a region with high DNAse I (open chromatin). For TP53, the deletion removes TAD boundaries, bringing the gene close to a cluster of eQTLs with high DNAse I (open chromatin).

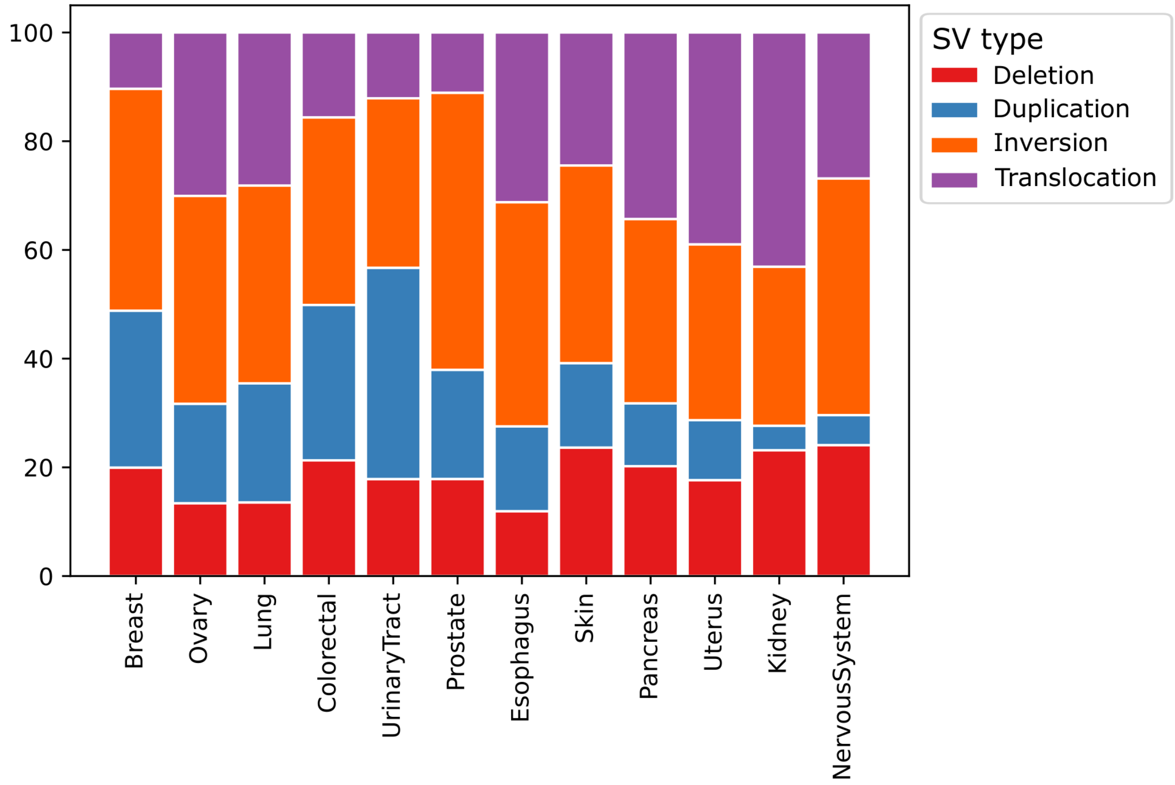

Fig. S7: **Relative contribution of each SV type to the predicted pathogenic SVs across all samples in each cancer type.**

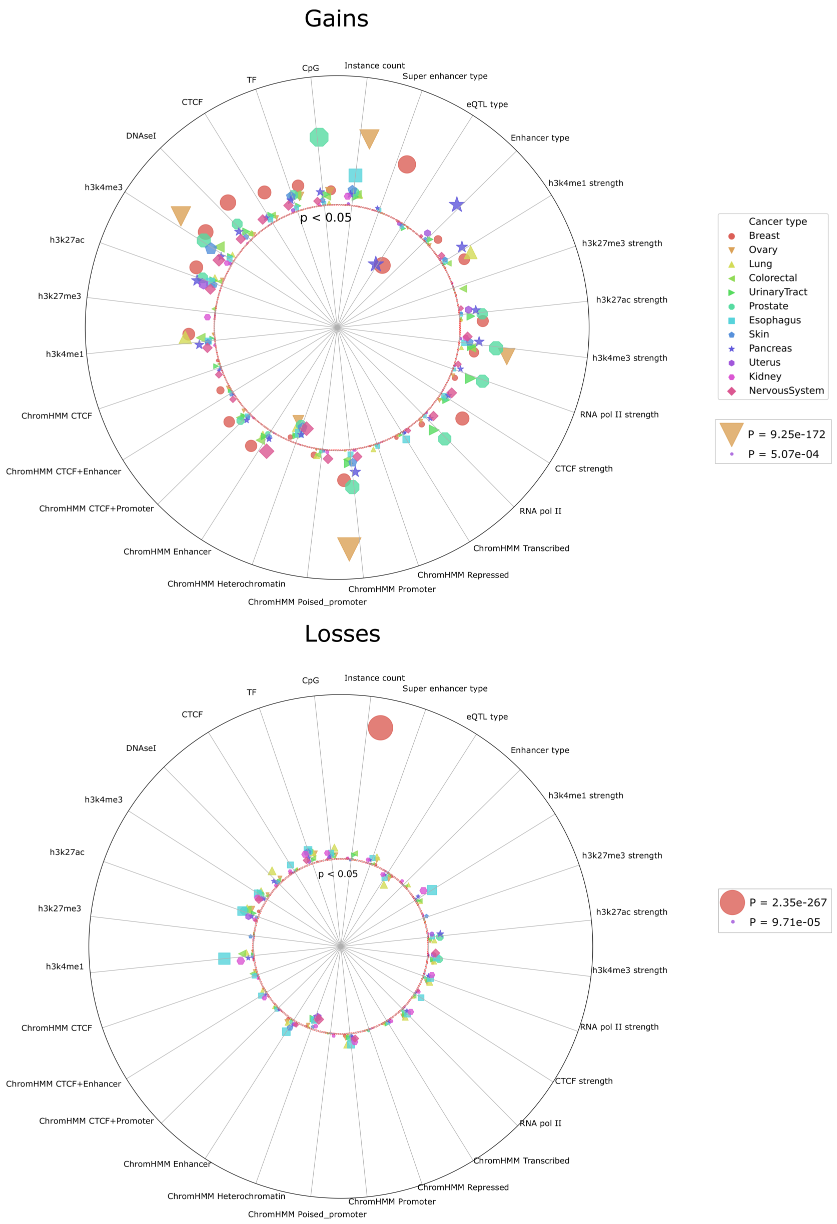

Fig. S8: **Regulatory elements affected by non-coding SVs, split into gains and losses. P-values are computed from a z-score based on the frequency of that feature in a gained regulatory element compared to 100 random gained regulatory elements. Points above the red dashed lines indicate P < 0.05 and z > 0, whereas points below the red dashed lines indicate P < 0.05 and z < 0. Note that the significances are slightly different from Fig 3, which is not split into gains and losses. In the majority of cancer types, (super) enhancers are affected rather than eQTLs, which is visible in combination with active enhancer marks (h3k27ac) and open chromatin (lack of ChromHMM heterochromatin). Gains reach higher significance as these are observed more often than by random chance. For losses, the number of lost regulatory elements per patient (instance count) stands out in breast cancer specifically.**

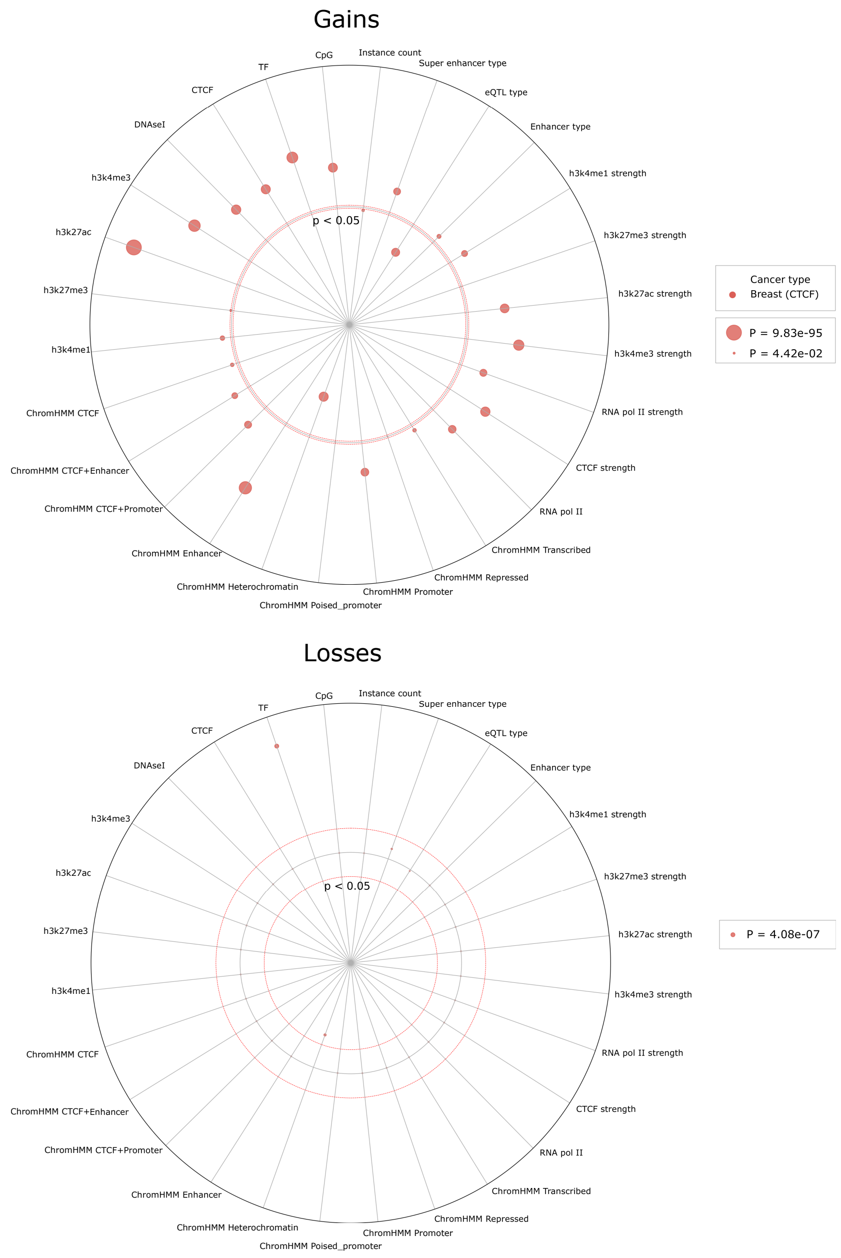

Fig. S9: **Regulatory elements affected by non-coding SVs, split into gains and losses, specific for using CTCF loops instead of TAD boundaries in breast cancer. P-values are computed from a z-score based on the frequency of that feature in a gained regulatory element compared to 100 random gained regulatory elements. Points above the red dashed lines indicate $P < 0.05$ and $z > 0$, whereas points below the red dashed lines indicate $P < 0.05$ and $z < 0$. Note that the significances are slightly different from Fig 5c, which is not split into gains and losses. The pattern of gaining (super) enhancers with active (h3k27ac) marks in open chromatin (lack of ChromHMM heterochromatin) is visible here too. Gains reach higher significance as these are observed more often than by random chance.**

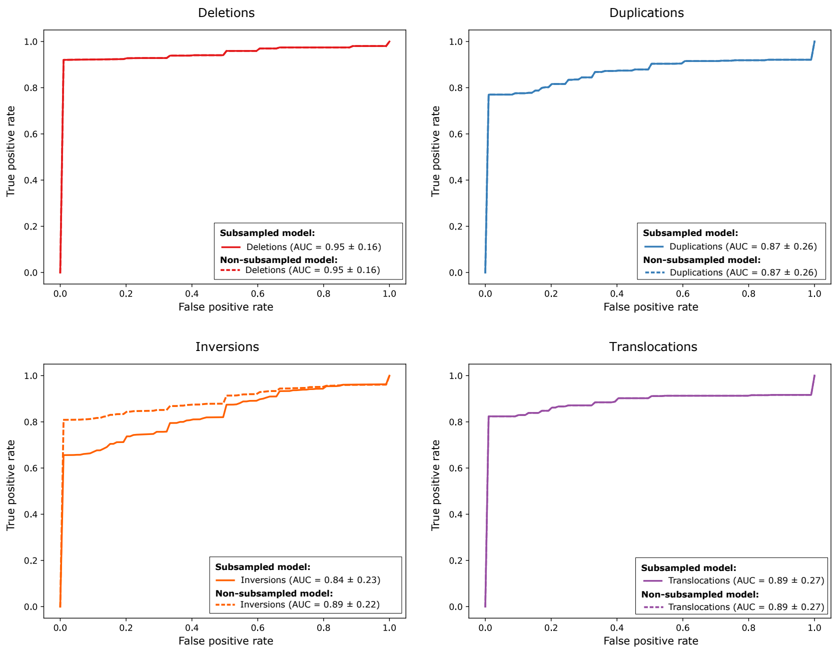

Fig. S10: **Subsampling bags does not significantly impact model performance. All performances are unaffected except for inversions, for which performance decreases only slightly.**
